# The Effect of Citalopram on Genome-Wide DNA Methylation of Human Cells

**DOI:** 10.1155/2018/8929057

**Published:** 2018-07-25

**Authors:** Riya R. Kanherkar, Bruk Getachew, Joseph Ben-Sheetrit, Sudhir Varma, Thomas Heinbockel, Yousef Tizabi, Antonei B. Csoka

**Affiliations:** ^1^Epigenetics Laboratory, Department of Anatomy, Howard University, 520 W St. NW, Washington, DC 20059, USA; ^2^Department of Pharmacology, Howard University, 520 W St. NW, Washington, DC 20059, USA; ^3^Tel-Aviv Brüll Community Mental Health Center, Clalit Health Services, 9 Hatzvi St., 6719709 Tel-Aviv, Israel; ^4^HiThru Analytics LLC, 1001 Spring St. No. 219, Silver Spring, MD 20910, USA

## Abstract

Commonly used pharmaceutical drugs might alter the epigenetic state of cells, leading to varying degrees of long-term repercussions to human health. To test this hypothesis, we cultured HEK-293 cells in the presence of 50 *μ*M citalopram, a common antidepressant, for 30 days and performed whole-genome DNA methylation analysis using the NimbleGen Human DNA Methylation 3x720K Promoter Plus CpG Island Array. A total of 626 gene promoters, out of a total of 25,437 queried genes on the array (2.46%), showed significant differential methylation (*p* < 0.01); among these, 272 were hypomethylated and 354 were hypermethylated in treated versus control. Using Ingenuity Pathway Analysis, we found that the chief gene networks and signaling pathways that are differentially regulated include those involved in nervous system development and function and cellular growth and proliferation. Genes implicated in depression, as well as genetic networks involving nucleic acid metabolism, small molecule biochemistry, and cell cycle regulation were significantly modified. Involvement of upstream regulators such as BDNF, FSH, and NF*κ*B was predicted based on differential methylation of their downstream targets. The study validates our hypothesis that pharmaceutical drugs can have off-target epigenetic effects and reveals affected networks and pathways. We view this study as a first step towards understanding the long-term epigenetic consequences of prescription drugs on human health.

## 1. Introduction

It has been hypothesized that pharmaceutical drugs can cause long-term epigenetic changes in the human genome [1, 2]. There is also evidence from animal models that antipsychotics can cause epigenetic changes [3] and that some drugs including antidepressants can interfere with the action of epigenetic enzymes, such as DNA methyltransferase 1 [4]. To test the hypothesis that pharmacological agents can change global DNA methylation in human cells, we chose a commonly used antidepressant, citalopram, and analyzed its effects on human cells by performing genome-wide DNA methylation analysis. Our hypothesis was that treatment with a typical pharmaceutical drug would cause widespread epigenetic changes. Confirmation of this hypothesis could have significant implications for the practice of medicine and for human health.

Citalopram belongs to the widely used class of antidepressant drugs called selective serotonin reuptake inhibitors (SSRIs) and is sold under the commercial name Celexa [5]. In terms of their mechanism of action, SSRIs prevent reuptake of the neurotransmitter serotonin (5-hydroxytryptamine (5-HT)) into the presynaptic cell, thereby increasing its bioavailability in the synaptic cleft, where it can bind to the postsynaptic receptors [6, 7]. Increasing the availability of serotonin in the synaptic cleft enhances serotonergic function and is believed to be responsible for alleviating depression-associated behavior [8]. By a different mechanism, SSRIs increase serotonin by downregulating presynaptic 5HT_1B_ autoreceptors (5-hydroxytryptamine or serotonin) that can otherwise inhibit serotonin release [9], thereby increasing synaptic serotonin availability [6]. While blocking reuptake of serotonin can increase its bioavailability and stimulate postsynaptic serotonin receptors to positively affect mood and anxiety, excessive firing of such serotonin-regulated neurons can negatively affect sleep, appetite, sexual function, and pain sensation, raising concerns regarding their adverse effects [6].

With the huge popularity of SSRIs and instances of controversial use in cases of “cosmetic psychopharmacology,” i.e., by individuals without clinical diagnoses, such side effects are of significant clinical concern [7]. Although earlier in their developmental phase they were considered to have fewer adverse effects than their first-generation counterparts (viz., tricyclic antidepressants), postmarketing clinical trials documented adverse effects mostly in terms of sexual dysfunction including anorgasmia, erectile dysfunction, genital anesthesia, and diminished libido in almost 75% of treated patients [6, 10–12]. Interestingly, these side effects appear to endure after treatment in some cases [13, 14], which is hard to explain using a standard pharmacological model.

A plausible cause of these persistent side effects is changes to the epigenome [1–3]. The epigenome of a cell is a unique, dynamic entity consisting of distinct DNA methylation patterns across gene enhancers, promoters, and bodies along with histone modifications that do not involve any changes to the actual DNA sequence. Recently, the effects of environmental factors, developmental processes, or lifestyle habits, such as diet and drugs, on modulation of gene expression via epigenetic modification have been studied in detail [15]. Epigenetic changes resulting from environmental effects such as traumatic life events can rewire neural circuits and alter neurotransmitter and endocrine systems resulting in stress-related psychiatric disorders such as major depression or posttraumatic stress disorder [15]. Based on this evidence, it can be posited that potential unknown mechanisms of action of SSRIs, as well as side effects, could be through epigenetic modification of genes [1, 2, 15].

For these reasons, its long history of use in depression treatment as well as its well-documented side effects including sexual dysfunction, sleep disturbances, and weight gain [6], citalopram was tested to assess its effect on genome-wide DNA methylation of human cells, with additional analysis on affected gene networks and signaling pathways, including but not limited to those implicated in neuropsychological function.

## 2. Materials and Methods

### 2.1. Cell Culture

Human embryonic kidney (HEK-293) cells were chosen for this study because they are used broadly for biomedical research, ranging from signal transduction to protein interaction studies, and are hence a good candidate for studying epigenetics as well. Advantages of using these cells over primary neurons is that they can be easily proliferated, maintained, preserved, and studied. Also, they express significant amounts of protein and mRNA for neurofilament (NF) subunits, such as NF-L, NF-M, NF-H, and *α*-internexin, as well as other neuron-specific proteins, suggestive of their neuronal lineage [16].

The HEK-293 cell line was purchased from ATCC and cultured in growth medium containing Dulbecco's Modified Eagle Medium (DMEM) (Life Technologies, CA, USA) supplemented with 10% fetal bovine serum (FBS) (Life Technologies, CA, USA) and 1x penicillin-streptomycin solution (Life Technologies, CA, USA) in a humidified incubator with 5% CO_2_ at 37°C. On reaching 90% confluence, cells were subcultured with a 1 : 6 split ratio in T25 flasks.

### 2.2. Cell Treatment

A toxicity curve was performed on the cells to determine the optimum concentration of citalopram hydrobromide (Sigma-Aldrich, MO, USA) that can be tolerated by the cells without changing their growth dynamics. Cells were cultured in growth media containing different concentrations of citalopram hydrobromide (10 *μ*M, 50 *μ*M, 90 *μ*M, 120 *μ*M, 160 *μ*M, and 200 *μ*M) for 48 hours. No effect was observed on cell growth kinetics or morphology below 120 *μ*M, but at concentrations above 160 *μ*M, an apoptotic-like cytotoxic effect was noted (Supplement 1). A 50 *μ*M solution of citalopram hydrobromide was determined to be the maximum concentration that could be safely used without any possibility of inducing any change in growth kinetics. HEK-293 cells in the treatment group (in triplicates) were cultured with 50 *μ*M citalopram hydrobromide for thirty days along with nontreated controls. All flasks were passaged and maintained under similar conditions as mentioned above for a period of thirty days.

### 2.3. DNA Extraction and MeDIP Chip Analysis

After a 30-day treatment, cells were lysed and genomic DNA was homogenized using QIAshredder (Qiagen) and extracted using the DNeasy kit (Qiagen) followed by sonication to generate fragments of about 200–1000 base pairs. Immunoprecipitation of methylated DNA was performed using Biomag™ magnetic beads coupled to a mouse monoclonal antibody against 5-methylcytidine. The immunoprecipitated DNA was eluted and purified by phenol-chloroform extraction and ethanol precipitation. The total input and immunoprecipitated DNA were labeled with Cy3- and Cy5-labeled random 9-mers, respectively, and hybridized to NimbleGen Human DNA Methylation 3x720K Promoter Plus CpG Island Arrays, which is a multiplex slide with 3 identical arrays per slide, and each array contains 27,728 CpG Islands annotated by UCSC and 22,532 well-characterized RefSeq promoter regions (from about −2440 bp to +610 bp of the Transcription Start Sites) totally covered by ~720,000 probes. Scanning was performed with the Axon GenePix 4000B Microarray Scanner by Arraystar Inc. (Rockville, MD, USA).

### 2.4. Data Normalization

Raw data was extracted as pair files by NimbleScan software. We performed median-centering, quantile normalization, and linear smoothing using NimbleScan by Nimblegen and R Bioconductor packages (Ringo, limma, and MEDME) [17]. The enrichment peaks and differentially methylated peaks were analyzed and annotated by NimbleScan software. The user guide and result data formats can be found at http://www.nimblegen.com/downloads/support/NimbleScan_v2p6_UsersGuide.pdf. After normalization, a normalized log2-ratio data (∗_ratio.gff file) was created for each sample. From the normalized log2-ratio data, a sliding-window peak-finding algorithm provided by NimbleScan v2.5 (Roche-NimbleGen) was applied to find the enriched peaks with specified parameters (sliding-window width: 750 bp; miniprobes per peak: 2; *p*-value minimum cutoff: 2; maximum spacing between nearby probes within peak: 500 bp). After obtaining the ∗_peaks.gff files, the identified peaks were mapped to genomic features: transcripts and CpG Islands.

### 2.5. Bioinformatics and Pathway Analysis of MeDIP Chip Results

*t*-tests and/or binomial tests were used to compute *p* values for differential methylation of CpG sites followed by multiple comparison correction of *p* values and computation of false detection ratio (FDR) using the Benjamini Hochberg method [18]. Genes that are significantly differentially methylated (*p* < 0.01) between the treated versus control groups were identified, and functional analysis of differentially methylated genes was performed using gene set enrichment analysis (GSEA). Gene promoters showing statistically significant changes in DNA methylation patterns were subjected to Ingenuity Pathway Analysis (IPA) (Ingenuity System Inc., CA, USA) for signaling pathway and gene network analysis. The *z*-scores predict activation states of transcriptional regulators and were calculated by an IPA-based algorithm (http://pages.ingenuity.com/rs/ingenuity/images/0812%20upstream_regulator_analysis_whitepaper.pdf).

## 3. Results and Discussion

### 3.1. Results

**Figure 1:**
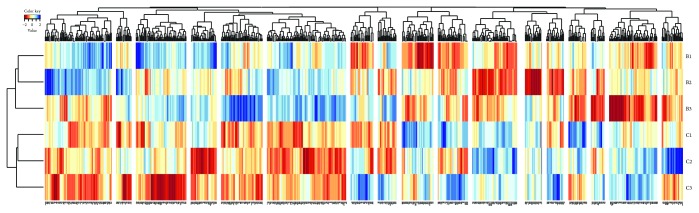
Heat map of hypermethylated and hypomethylated gene promoters. This heat map represents differentially methylated gene promoters between citalopram-treated (B1, B2, and B3) and control (C1, C2, and C3) samples with significant values from MeDIP chip analysis, grouped into clusters. The scale represents hypermethylated gene promoters (values 0 to +2) in blue and hypomethylated gene promoters (values 0 to −2) in red. Each column represents a gene, as specified on the gene axis at the bottom, that is either downregulated (hypermethylated promoters in blue) or upregulated (hypomethylated promoters in red) between samples represented in the six rows. The right axis represents overall methylation clustering between treated and control samples, and the top axis represents quantitative methylation clustering between significant genes.

Genome-wide DNA methylation analysis revealed that citalopram causes significant differential methylation (*p* < 0.01) in 626 gene promoters (from about −2440 bp to +610 bp of the transcription start sites) compared to controls (2.46%). Overall, there were more gene promoters hypermethylated (354; 1.39%) than hypomethylated (272; 1.07%) (Supplement 2a). Means and standard deviations for all of the samples can be seen in Supplement 2b. A heat map (Figure 1) represents differential DNA methylation between treated (B1, B2, and B3) and control (C1, C2, and C3), grouped into clusters. Since our analysis only included significant gene promoters without intragenic and intergenic regions, we were able to translate our methylation data into gene expression data for IPA without complication; hypermethylated promoters representing downregulation and hypomethylated promoters representing upregulation of gene expression, by default. We assigned positive and negative values to peak differential methylation values to correlate to upregulation or downregulation of gene expression, respectively (Supplement 3). Hereafter, we refer to these gene promoters as genes for simplicity and refer to activation from gene induction as upregulation and inhibition from gene silencing as downregulation.

**Figure 2:**
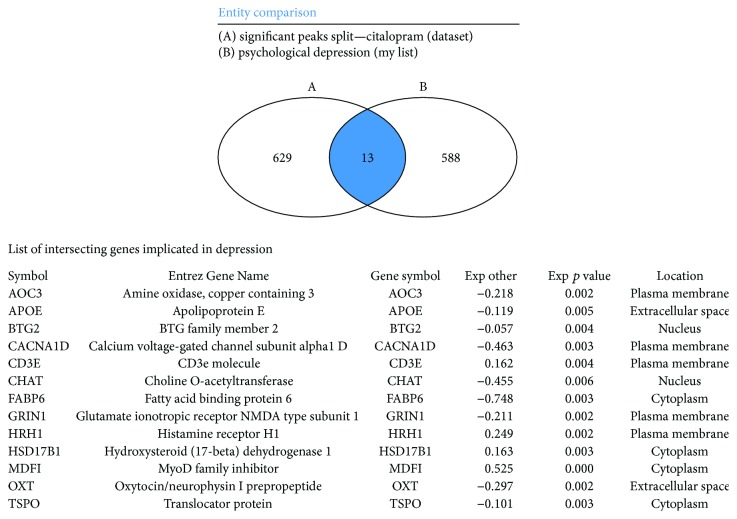
Venn diagram of genes involved in depression with significant differential methylation. This figure shows a set of 13 genes identified from our dataset with significant differential methylation resulting from citalopram treatment overlapping with a curated list of genes implicated in psychological depression according to the current IPA database, shown in Venn diagram form. Genes are classified according to their subcellular location, *p* value, and expression value where positive expression values indicate upregulation and negative expression values represent downregulation. A few important genes included in this list are OXT, GRIN1, CHAT, and CACNA1D which are potential regulators of neurophysiological processes and have a high degree of implication in psychological disorders.

We especially wanted to analyse any differential methylation caused by citalopram at genes that are either a part of the epigenetic modifier groups or involved in depression-related behavior. We compared our dataset with a curated list of a total 601 genes and molecules implicated in psychological depression (IPA) and found 13 genes common showing significant differential methylation (Figure 2, Supplement 4), including BTG2, FABP6, GRIN1, HRH1, HSD17B1, MDFI, OXT, and TSPO. We also found six epigenetic enzymes with significant differential methylation, including HDAC6, SET, SETBP1, SETD82, SIRT1, and TDG (Supplement 5).

**Figure 3:**
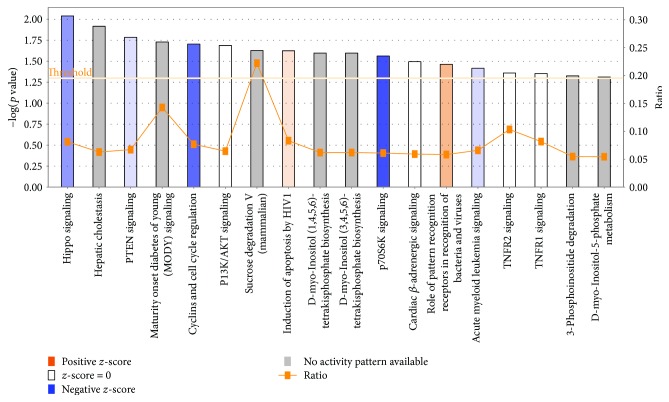
Top canonical pathways altered by citalopram. This bar graph enlists the top canonical pathways predicted to be altered by citalopram treatment using ingenuity pathway core analysis. Citalopram treatment results in differential methylation of significant genes from our dataset that are enriched in canonical pathways, like Hippo signaling, PTEN signaling, maturity-onset diabetes of the young (MODY) signaling, and cyclins and cell cycle regulation signaling based on their *z*-score, ratio, and −log (*p* value). A positive *z*-score (orange) denotes activation of pathway (e.g., role of pattern recognition receptors in recognition of bacteria and viruses), and a negative *z*-score (blue) denotes inhibition of a pathway (e.g., P70S6K signaling). The ratio (orange line with blocks) represents a ratio of genes from our dataset that is enriched in the pathway divided by the total number of genes enriched in the same pathway according to current IPA database [e.g., 22.5% sucrose degradation V (mammalian)]. Threshold is set at the lowest level of confidence that is acceptable statistically (*p* < 0.05).

**Figure 4:**
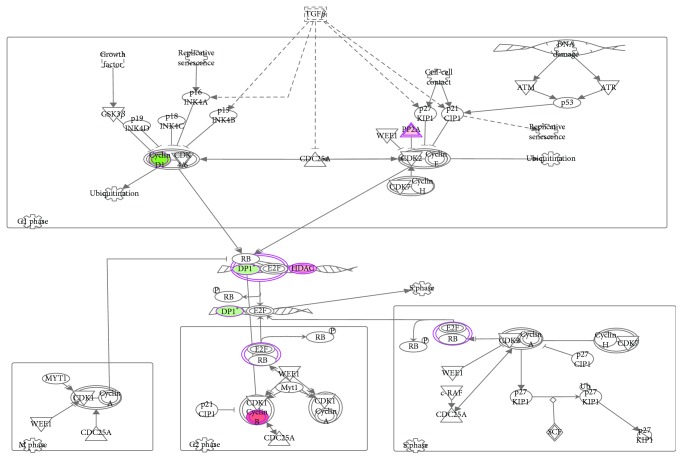
Cyclins and cell cycle regulation is the top pathway downregulated by citalopram treatment. This figure represents the cyclins and cell cycle regulation pathway as the top signaling pathway downregulated with a significant *p* value (*p* value = 1.98*E* − 02) due to citalopram treatment. IPA identifies this pathway as it involves differentially methylated genes from our dataset like cyclin B, HDAC, and P2A that are upregulated (red) and cyclin D1 and Dp1 that are downregulated (green). The color intensity is proportional to the extremity of upregulation or downregulation.

In a broad analysis of canonical signaling pathways, gene networks and biological functions using IPA's core analysis function, we found that significant genes from our dataset were enriched in canonical pathways including Hippo signaling (*p* value = 9.14*E* − 03), PTEN signaling (*p* value = 1.64*E* − 02), maturity-onset diabetes of the young (MODY) signaling (*p* value = 1.87*E* − 02), and cyclins and cell cycle regulation signaling (*p* value = 1.98*E* − 02) (Table 1, Figures 3 and 4). These also included inflammation-related signaling pathways like TNFR2 (*p* value = 4.37*E* − 02) and TNFR1 (*p* value = 4.43*E* − 02) (Table 1). Many of these pathways show overlapping patterns (Supplement 6). The chief associated gene network functions of the canonical pathways include nucleic acid metabolism, small molecule biochemistry, and cell signaling associated with a number of diseases including cancer and nervous system dysfunction (Table 1). Genes are enriched for molecular and cellular functions including protein synthesis, cellular movement, and drug metabolism (Table 1). Novel regulatory networks involving CASZ1 in quality of metal ion and miR 199a-5p in growth of plasma membrane projections were identified (Supplement 7). Thus, a wide variety of gene networks and pathways were affected by the citalopram treatment.

**Figure 5:**
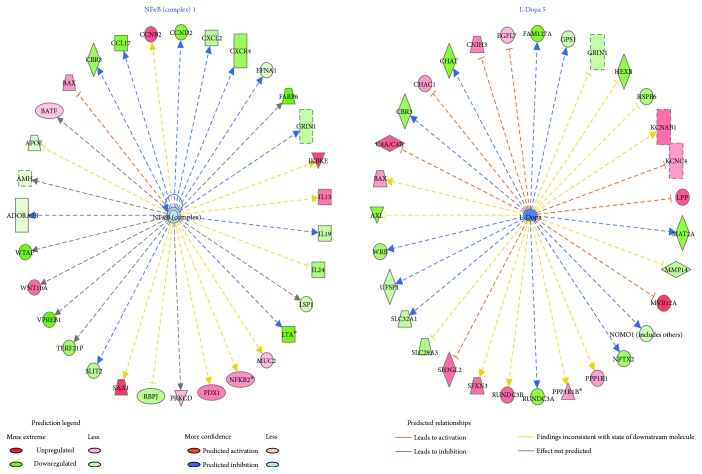
NF*κ*B complex 1 and L-dopa 5 identified as upstream regulators with predicted inhibition. This figure represents the two upstream regulators with predicted significant differential regulation resulting from citalopram treatment based on the methylation states (hypermethylated or hypomethylated) of their downstream targets by ingenuity pathway upstream regulator analysis. NF*κ*B complex 1 is a transcription factor predicted with a higher degree of inhibition (green) with *p* value = 1.79*E* − 04. L-Dopa, the initial substrate for neurotransmitter dopamine, is also predicted to be inhibited (green) at a lower degree with *p* value = 1.22*E* − 03. With upstream regulators NF*κ*B complex 1 and L-dopa at the center, the dotted lines with arrow indicate downstream target genes that are upregulated (red) or downregulated (green) due to differential methylation as indicated in our dataset. A dashed line means indirect interaction, continuous line means direct interaction, line with arrow means “acts on” and line with bar at the end means “inhibits.” These basic relationships between molecules represented in the figure are based on literature-reported effects, and the color coding represented in the legend is used for correlation of known relationships with observed gene expression effects resulting from treatment of citalopram. For example, based on literature, L-dopa indirectly acts on SLC32A1, a polyamine transporter. However, in the presence of citalopram, L-dopa bioavailability is decreased, and it is predicted to have an inhibitory effect on SLC32A1 indirectly such that it is downregulated.

Next, we analyzed the main upstream regulators predicted for differential regulation based on their downstream target states (hypermethylated or hypomethylated) and found that citalopram most importantly affected the NF*κ*B complex (*p* value = 1.79*E* − 04) and L-dopa pathway (*p* value = 1.22*E* − 03) (Figure 5). Other significant upstream regulators with predicted differential regulation include FSH (*p* value = 4.22*E* − 02); BDNF (*p* value = 2.23*E* − 02); IL13 (*p* value = 8.92*E* − 03); PRKCD, a protein kinase C (*p* value = 4.64*E* − 01); and GLI1, a Kruppel family member of zinc finger proteins (*p* value = 3.99*E* − 01).

Finally, top physiological systems affected by citalopram identified by IPA included nervous system development diseases and function with 24 genes involved in neurotransmission (*p* value = 1.25*E* − 02), 23 genes related to outgrowth of neurites (*p* value = 1.59*E* − 02), 9 genes related to excitatory postsynaptic potential (*p* value = 1.48*E* − 02), 6 genes related to quantity of synapse (*p* value = 1.21*E* − 02), and 3 genes related to loss of dendritic spines (*p* value = 1.42*E* − 02). Additionally, 47 genes related to morphology of the nervous system (*p* value = 1.95*E* − 02), 35 genes related to development of the central nervous system (*p* value = 1.45*E* − 02), 21 genes related to sensation (*p* value = 3.67*E* − 03), 11 genes related to development of the cerebral cortex (*p* value = 1.20*E* − 02), 8 genes related to abnormal morphology of the hippocampus (*p* value = 1.72*E* − 02), 7 genes related to abnormal morphology of the synapse (*p* value = 2.96*E* − 03), and 3 genes related to development of the hypothalamus (*p* value = 1.50*E* − 03) were identified (Supplement 8). These results, in particular, were interesting because of the known mechanism of citalopram action on the nervous system-based serotonin transporter, along with unknown targets affected by epigenetic mechanisms, that can be further delineated in the future.

### 3.2. Discussion

We have previously outlined a potential mechanism for understanding the direct and indirect effects of environmental factors, including pharmaceutical drugs [1, 15] on the epigenome. Here, we attempted to confirm the hypothesis that pharmacological agents can cause permanent changes via epigenetic reprogramming. The results show that our first test drug, citalopram, can cause genome-wide DNA methylation alterations as revealed by significant differential methylation in hundreds of genes, as well as predicted impact on signaling pathways and/or physiological systems, some of which are described below.

#### 3.2.1. Reproductive and Sexual Function

The OXT gene, producing oxytocin, is downregulated by citalopram. Since oxytocin plays a significant role in parturition and milk ejection and is also implicated in cognition, tolerance, adaptation, and complex sexual and maternal behavior, its downregulation by SSRIs may be one of the underlying causes of sexual dysfunction seen in many cases [12, 19]. In terms of upstream regulators, inhibition of the dopa pathway, involved in the synthesis of dopamine, also coincides with the numerous findings of negative effects of SSRIs on dopaminergic signaling including sexual dysfunction [20].

Amongst other upstream regulators, we saw predicted inhibition of follicle-stimulating hormone (FSH), which is responsible for maturation of ovarian follicles in females and spermatocytes in males. FSH is regulated by gonadotropin-releasing hormone (GnRH), also included in one of our gene networks, affecting functions including cell signaling, molecular transport, and vitamin and mineral metabolism (Supplement 8). Previous studies have confirmed the side effects of SSRIs on reproductive and neuroendocrine dysfunction in wildfish involving changes in ovarian and hypothalamic gene expression, spermatogenesis, and sex steroid production [21–23]. In a month-long treatment of male zebrafish with citalopram, different stages of spermatogenesis were inhibited, whereas short-term treatment downregulated the expression of GnRH and serotonin-related genes TPH2 and SERT [10]. Moreover, SSRIs affect the hypothalamic-pituitary-testis (HPT) axis in depressed male patients suffering from SSRI-induced sexual dysfunction due to significantly lower serum levels of luteinizing hormone (LH), FSH, and testosterone [24, 25]. These studies and our current data imply that the imbalances in GnRH, FSH, and LH production associated with abnormal serotonin levels might be epigenetic at source and at least partly responsible for SSRI-induced sexual and reproductive dysfunction.

#### 3.2.2. Signaling Pathways: Molecular and Metabolic Interference

Primary pathways such as Hippo signaling, PTEN signaling, and cyclins and cell cycle regulation signaling were downregulated. The Hippo signaling pathway regulates organ size control, tumor suppression, tissue regeneration, and stem cell self-renewal [26]. Cyclins and cyclin-dependent kinase (CDK) family members are involved in a range of diverse functions including transcription, DNA damage repair, proteolytic degradation, epigenetic regulation, metabolism, stem cell self-renewal, neuronal functions, and spermatogenesis [27]. PTEN is a tumor suppressor, and modification of PTEN signaling networks results in manifestation of developmental defects and increased risk of cancer [28]. Thus, inhibition or dysregulation of signaling pathways may increase risk of cancer [29, 30]. Another interesting finding is the involvement of pathways for maturity-onset diabetes of the young (MODY). Previous studies report significant weight gain, insulin resistance, and worsening glycemic control as side effects of chronic SSRI usage [31].

#### 3.2.3. Neurological and Psychiatric Pathways

The translation of early life stress into major depressive disorders in adulthood is possibly rooted in epigenetic alteration of candidate genes, including the serotonin transporter (SLC6A4), via DNA methylation, histone acetylation and methylation, and miRNAs, which also is a mode of therapeutic action of some antidepressant drugs [32–34]. We identified 13 genes associated with depression-related disorders that were differentially methylated by citalopram, which in some ways seems to be quite a low number considering the therapeutic target. In any case, B-cell translocation gene 2 (BTG2), reported to be upregulated (in the prefrontal cortex) in major depression, was downregulated [35]. Additionally, the MyoD family inhibitor (MDFI) that is downregulated in depression (dorsolateral prefrontal cortex) was upregulated [36]. Citalopram also downregulated translocator protein (TSPO), generally upregulated in depression [37]. In a study using a rat model involving long-term treatment of depression with escitalopram (a stereoisomer of citalopram), p11, a calcium-binding protein, generally downregulated in depression, was induced by specific hypomethylation of the p11 gene promoter, increasing gene expression and reversing depression-like behavior [38, 39]. Other genes including FABP6 (fatty acid binding protein 6), downregulated in the prefrontal cortex in major depression [35]; GRIN1 (glutamate ionotropic receptor NMDA subunit), implicated in stress-related psychiatric disorders [40]; HRH1 (histamine receptor H1), known to be blocked by TCAs [41]; and HSD17B1 (hydroxysteroid 17-beta dehydrogenase 1), associated with female depression [42], were likewise differentially methylated. These mechanisms indicate unique effects of SSRIs and suggest novel therapeutic targets for treatment of depression.

#### 3.2.4. Inflammation

Inflammation-related upstream regulators like the NF*κ*B complex are inhibited and IL13 activated. Inflammation plays an important role in the pathophysiology of depression as seen in many patients with elevated proinflammatory cytokine levels [43]. Modulation of inflammatory networks by antidepressants has previously been associated with decreased inflammation in male patients using SSRIs but, curiously, increased inflammation in patients using other types of antidepressants [44]. However, it should be noted that specific interactions between innate and adaptive immune systems and neurotransmitters and neuronal circuits may influence risk for depression and response to antidepressants [45, 46].

#### 3.2.5. Nervous System Development and Function

Nervous system development and function (*p* value = 1.95*E* − 02 to 2.67*E* − 04) was one of the systems most significantly affected by the treatment (Table 1). 47 genes related to morphology of the nervous system (*p* value = 1.95*E* − 02), 35 genes related to development of the central nervous system (*p* value = 1.45*E* − 02), and 11 genes related to development of the cerebral cortex (*p* value = 1.20*E* − 02) were identified. These effects may be related to changes in autonomic functions (e.g., tachycardia), hypothermia, and changes in mental status (e.g., agitation, anxiety, and confusion) [47]. In mice, increased serotonergic activity postnatally can propagate abnormal neuroanatomical development of the somatosensory cortex along with functional response deficits [48]. Intrauterine antidepressant exposure can cause epigenetic changes affecting neonatal development and health [49] and lasting abnormal emotional behaviors [48, 49]. Thus, epigenetic changes at genetic loci involved in neuroanatomical development have major implications on the use of SSRIs to treat depressive behaviors.

One potential limitation with this pilot study is that HEK-293 cells have not been shown to express high levels of the serotonin transporter, SERT, nor been shown to synthesize high amounts of serotonin in the extracellular medium, compared to neurons. Hence, in this case, we argue that the effects of citalopram seen on DNA methylation in these cells are more likely to be 5HT-independent. HEK-293 cells *are* known to abundantly express a diverse repertoire of receptors such as *β*2-adrenergic, muscarinic acetylcholine, sphingosine-1-phosphate, P2Y1 and P2Y2, corticotropin-releasing factor type 1, and somatostatin- and thyrotropin-releasing hormone receptors, and citalopram has been shown to interact strongly with some of these receptors, in the concentration range used in this study, so it may well be eliciting epigenetic effects through these pathways. Moreover, the data is consistent with our initial hypothesis that the epigenetic effects of chemicals could be both direct (acting directly on DNA or DNA-modifying enzymes) and indirect (acting through receptors or signaling pathways) [1, 15], in which case a direct effect on SERT is not necessary to induce epigenetic changes. It is also possible that in the presence of 5-HT and SERT, we may see different epigenetic effects of citalopram from those observed in the HEK-293 cells. In any case, we intend to repeat this experiment using primary human neurons as the target cells, rather than a proliferating cell line, in order to gain greater insights into potential *in vivo* effects.

## 4. Conclusions

### 4.1. Whole-Genome Epigenetic Analysis as an Aspect of the Drug Development Process

In this study, we wanted to explore, in an initial investigative pilot experiment, the potential for a typical, widely used pharmaceutical drug to cause epigenetic changes in human cells, both beneficial and potentially harmful. We used human genome-wide promoter methylation analysis to delineate unique gene methylation profiles arising from short-term treatment with citalopram. These results could serve as proof of principle for such assays to become standard protocol during the toxicological analysis stage of drug development, from bench to bedside. Such epigenetic toxicological analysis could eventually revolutionize the safety of personalized medicine. We view this paper as an initial first step in a much broader inquiry into the epigenetic mechanisms of pharmacological agents.

### 4.2. Drugs and Waddington's Canal

We also wanted to explore the possibility that the magnitude of the epigenetic changes caused by a typical drug is enough to displace a cell from its normal “groove” in the “epigenetic landscape.” This is a term derived from the original work by C. H. Waddington and represents a series of branching valleys depicting developmental pathways and ridges between valleys that are barriers to transitions between steady cellular states that reside in the valleys [50]. Waddington also coined the term “canalization,” meaning that, up to a certain threshold, any genetic variation or environmental insult to a cell will be nullified and the cell will remain within its groove, but above this threshold, the cell would flip over into an adjacent pathway or “valley” [51]. A modern example of altering canalization is the phenomenon of reprogramming somatic cells to pluripotency, which is achieved by activating epigenetic switches and driving a cell back up its lineage to the highest point in the landscape via the reversal of differentiated gene expression to a fully embryonic-like state [52]. Interestingly, such total reprogramming of differentiated cells to pluripotency can now be achieved by the use of small molecules alone [53]. Therefore, we reasoned that if a chemical cocktail alone is capable of reversing a cell's lineage, then there is also a possibility that pharmaceutical drugs in isolation or in combination (as in polypharmacy) can alter cells' epigenetic profiles sufficiently that they are no longer in their original differentiated state. It is highly unlikely that this would represent a recanalization event per se but rather a slight “shift” in the groove causing marginal dysdifferentiation.

### 4.3. The Concept of “Pharmaceutical Reprogramming”

As stated, it is likely that the epigenetic effects of citalopram are much too weak to induce phenotypic conversion or alter lineage but may be just robust enough to cause a partial dysdifferentiation event, whereby a cell's location in its epigenetic landscape is marginally altered. Such a differentiation “wobble” would result from all of the changes in DNA methylation altering the cell's normal biochemistry. We have termed this partial dysdifferentiation from pharmacological exposure “pharmaceutical reprogramming.” Pharmaceutical reprogramming could affect cells and tissues at the submicroscopic level but might not be evident microscopically or macroscopically. It will be important to explore this hypothesis further in future studies, in order to better understand the epigenetic effects of drugs capable of affecting cellular function and integrity. The implications of these findings, if true, could have enormous importance for human health.

## Figures and Tables

**(a):** 

Top canonical pathways		
Name	*p* value	Overlap
Hippo signaling	9.14*E* − 03	8.1% 7/86
Hepatic cholestasis	1.21*E* − 02	6.3% 10/159
PTEN signaling	1.64*E* − 02	6.7% 8/119
Maturity-onset diabetes of the young (MODY)	1.87*E* − 02	14.3% 3/21
Cyclins and cell cycle regulation	1.98*E* − 02	7.7% 6/78

**(b):** 

Top upstream regulators	
Upstream regulator	*p* value of overlap
NS-398	5.54*E* − 05
NF*κ*B (complex)	1.79*E* − 04
ACKR3	3.72*E* − 04
RP 73401	8.44*E* − 04
L-Dopa	1.22*E* − 03

**(c):** 

Top diseases and biofunctions		
Name	*p* value	Number of molecules
*Diseases and disorders*		
Cancer	2.00*E* − 02 to 1.20*E* − 06	511
Organismal injury and abnormalities	2.00*E* − 02 to 1.20*E* − 06	515
Hypersensitivity response	5.02*E* − 04 to 5.02*E* − 04	7
Dermatological diseases and conditions	1.97*E* − 02 to 6.53*E* − 04	25
Immunological disease	1.95*E* − 02 to 6.53*E* − 04	22

*Physiological system development and function*		
Lymphoid tissue structure and development	2.12*E* − 02 to 7.93*E* − 05	56
Tissue morphology	2.12*E* − 02 to 7.93*E* − 05	107
Humoral immune response	1.37*E* − 02 to 1.84*E* − 04	28
Connective tissue development and function	2.12*E* − 02 to 2.67*E* − 04	42
Nervous system development and function	1.95*E* − 02 to 2.67*E* − 04	107

**(d):** 

Top networks		
ID	Associated network functions	Score
(1)	Nucleic acid metabolism, small molecule biochemistry, cell signaling	42
(2)	Auditory disease, cancer, cardiovascular disease	42
(3)	Carbohydrate metabolism, drug metabolism, small molecule biochemistry	37
(4)	Cellular growth and proliferation, tissue development, cellular movement	37
(5)	Embryonic development, humoral immune response, lymphoid tissue structure and development	33

This is a comprehensive list of the top canonical pathways and top upstream regulators with predicted differential regulation as well as diseases, biofunctions, and networks with the highest enrichment involving significant genes identified in our dataset based on *p* values and other criteria set by IPA. Amongst the top canonical pathways, Hippo signaling has the highest overlap in genes from our dataset that are enriched in the pathway divided by the total number of genes enriched in the Hippo pathway, that is, 8.1% according to the current IPA database. NS-398 which is a cyclooxygenase inhibitor is amongst the top upstream regulator with a *p* value of 5.54*E* − 05. The top diseases associated with citalopram treatment include cancer with 511 molecules predicted to have differential regulation, whereas the top physiological system development and function predicted to be effected includes nervous system development and function with 107 molecules identified by IPA. The top associated gene network functions include nucleic acid metabolism, small molecule biochemistry, and cell signaling with a score of 42 generated by the IPA algorithm. A score of 50 is considered as high and below 20 is low. Predicted activation.

## Data Availability

Complete data files are available on request.

## Supplementary Materials

Supplementary 1 Supplement 1: images of HEK-293 cells in increasing concentrations of citalopram from 120 *μ*M to 200 *μ*M. No effect was observed on cell growth kinetics or morphology below 120 *μ*M, but at a concentration above 160 *μ*M, an apoptotic-like cytotoxic effect was noted.

Supplementary 2 Supplement 2a: table with a list of significantly differentially methylated peaks (*p* < 0.01) that lie within 2000 base pairs of the transcription start site of a gene, generated using the initial raw data. All coordinates were transformed from hg18 to hg38, and the genes were reannotated. Peaks were remapped to the latest genome (hg38) and refiltered according to distance from the nearest gene TSS. The “Direction” column indicates which way the differential methylation goes (hypermethylated in treated or hypomethylated). The “Peak differential methylation” column (col M) shows the average difference in methylation level between treated and untreated (positive values are higher methylation in treated compared to untreated; negatives are lower). There are more peaks hypermethylated in treated than hypomethylated (354 hypermethylated peaks versus 272 hypomethylated peaks). Each peak lies within 2000 base pairs of one or more genes. The list of genes is in column C and the distance of their TSS from the middle of the peak is in column L. Positive numbers indicate that the peak middle is upstream of the TSS; negative numbers indicate that the peak middle is downstream. Supplement 2b: means and standard deviations for all of the significant peaks.

Supplementary 3 Supplement 3: table with list of significantly differentially methylated peaks (*p* < 0.01) that lie within 2000 base pairs of the transcription start site of a gene, used as raw data for Ingenuity Pathway Analysis. Hypomethylated gene promoters with significantly differentially methylated peaks were assigned positive signs and hypermethylated gene promoters were assigned negative signs as opposed to the list in Supplement 1, to correlate with upregulation or downregulation of gene-expression, respectively.

Supplementary 4 Supplement 4: table listing all genes implicated in psychological depression curated by current IPA database. Information regarding gene/molecule and their respective Entrez Gene IDs for human, mouse, and rat are specified. This list was used for searching any common genes in our list of significant genes as obtained after MeDIP chip analysis on citalopram-treated human cells.

Supplementary 5 Supplement 5: table listing all epigenetic modifiers according to the current IPA database. This list was used for searching any common genes encoding epigenetic enzymes in our list of significant genes as obtained after MeDIP chip analysis on citalopram-treated human cells.

Supplementary 6 Supplement 6: overlapping between individual significant canonical pathways identified by IPA that are altered by citalopram treatment. Each node represents one canonical pathway, and each link represents a set of genes acting between two pathways determined by Fisher's exact test *p* value. Darker red shade of nodes represents highly significant pathways and lighter shade of red represents less significant ones. Line width of links corresponds to the number of molecules shared between two pathways where no line means no shared molecules between two pathways and blue line means strong overlap of molecules between canonical pathways.

Supplementary 7 Supplement 7: this figure represents a novel regulatory network identified by IPA. SET as predicted to be upregulated in our dataset can be involved in regulation of growth of plasma membrane projections in addition to miR 199a-5p, SIRT, and DDR1. Red color represents upregulation. Lighter red represents activation, and blue represents inhibition. Red line denotes activation, yellow line denotes finding inconsistencies, and black line denotes effect not predicted.

Supplementary 8 Supplement 8: an all-inclusive list of nervous system development-, function-, and disease-related genes based on significant genes from our dataset as identified by IPA analysis of citalopram-treated human cells. Each function with *p* value and activation *z*-score, gene/molecule names, and total number of genes/molecules with predicted differential regulation from citalopram treatment are listed.
