# Supplementary figures and images for: The Effect of Citalopram on Genome-Wide DNA Methylation of Human Cells
Source: Int J Genomics. 2018 Jul 25;2018:8929057. doi: 10.1155/2018/8929057 (PMC6083487; doi:10.1155/2018/8929057)

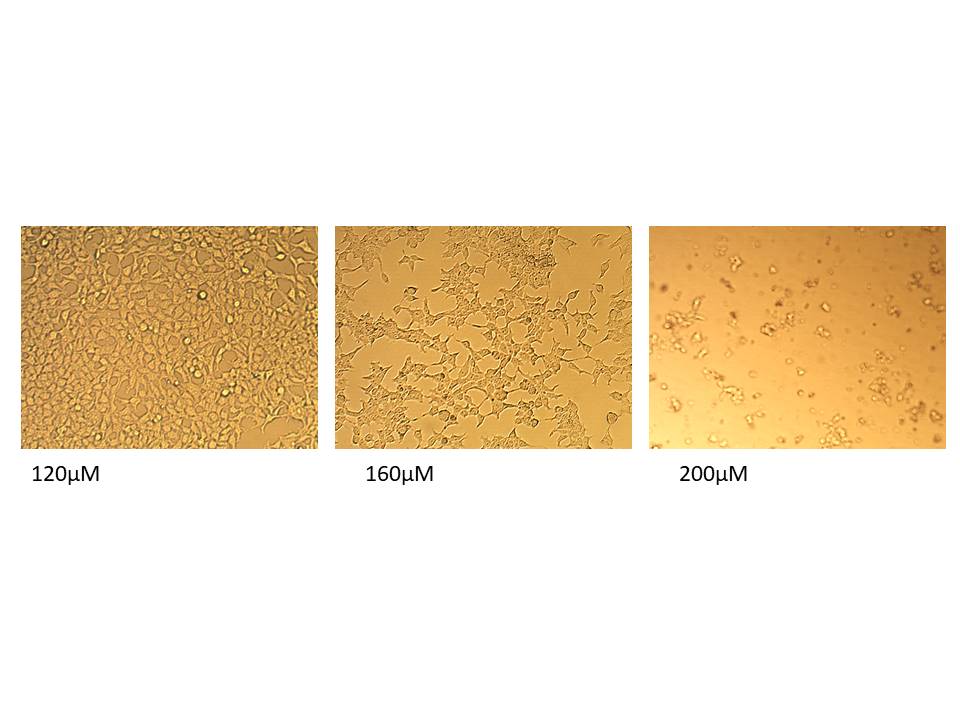

Supplement: Supplementary 1 — Supplement 1: images of HEK-293 cells in increasing concentrations of citalopram from 120 μM to 200 μM. No effect was observed on cell growth kinetics or morphology below 120 μM, but at a concentration above 160 μM, an apoptotic-like cytotoxic effect was noted. [file 8929057.f1.jpg]

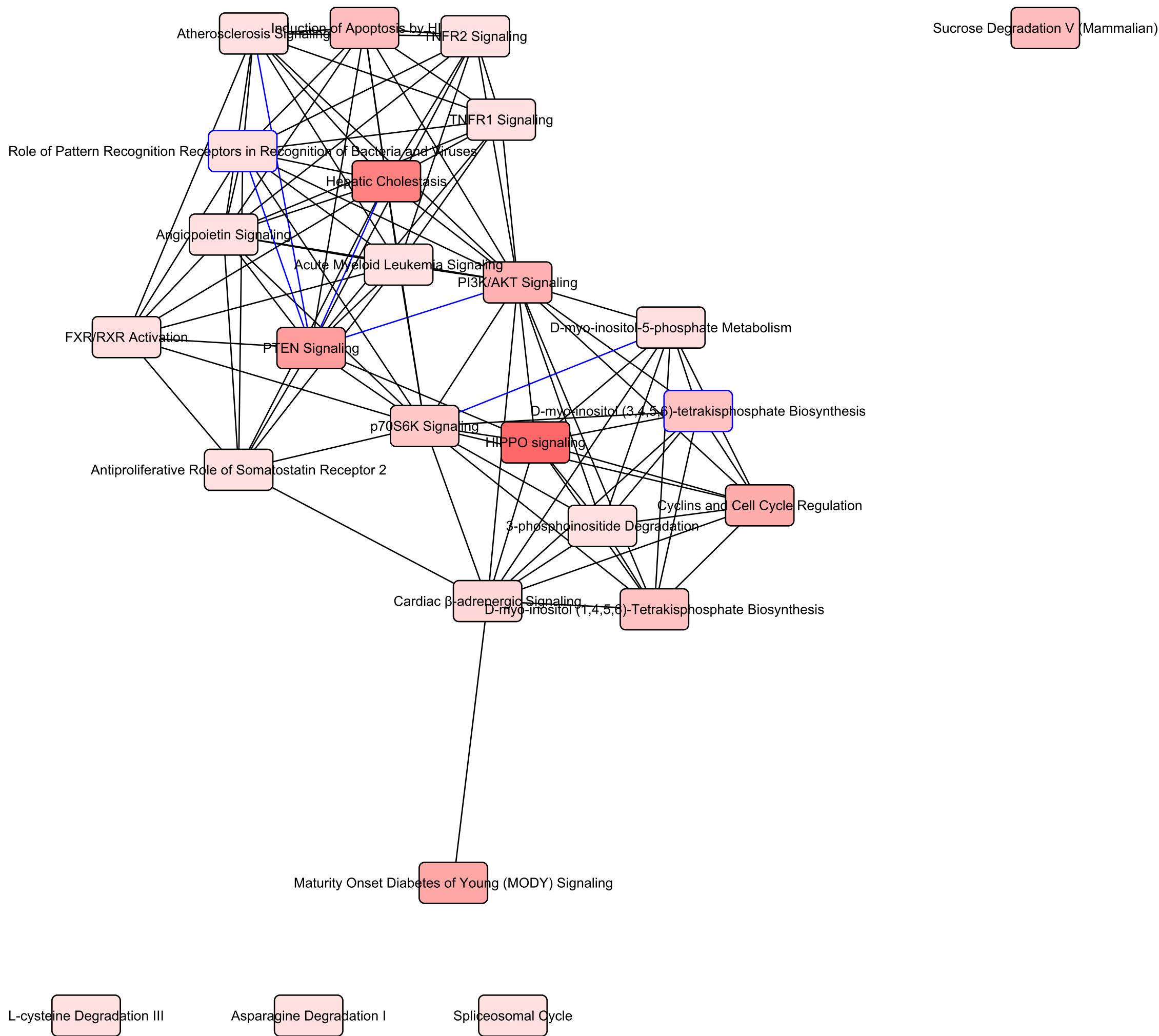

Supplement: Supplementary 6 — Supplement 6: overlapping between individual significant canonical pathways identified by IPA that are altered by citalopram treatment. Each node represents one canonical pathway, and each link represents a set of genes acting between two pathways determined by Fisher's exact test p value. Darker red shade of nodes represents highly significant pathways and lighter shade of red represents less significant ones. Line width of links corresponds to the number of molecules shared between two pathways where no line means no shared molecules between two pathways and blue line means strong overlap of molecules between canonical pathways. [file 8929057.f6.pdf]

miR-199a-5p (and other miRNAs w/seed CCAGUGU)

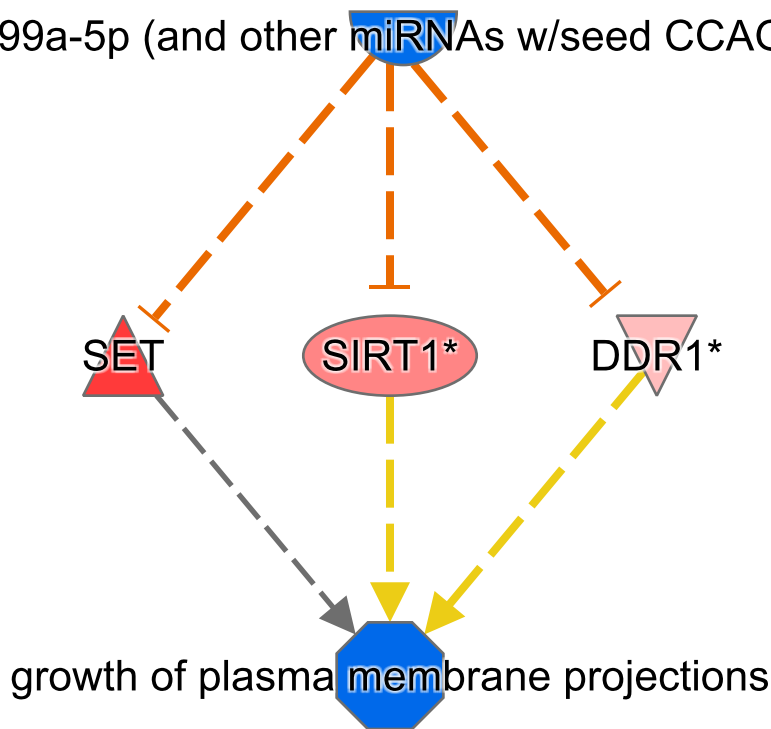

Supplement: Supplementary 7 — Supplement 7: this figure represents a novel regulatory network identified by IPA. SET as predicted to be upregulated in our dataset can be involved in regulation of growth of plasma membrane projections in addition to miR 199a-5p, SIRT, and DDR1. Red color represents upregulation. Lighter red represents activation, and blue represents inhibition. Red line denotes activation, yellow line denotes finding inconsistencies, and black line denotes effect not predicted. [file 8929057.f7.pdf]
